# Mechanochemical Synthesis of MgV_2_O_4_: Reactivity Pathways Driven by Milling Energy and Precursors

**DOI:** 10.1021/acs.inorgchem.5c03095

**Published:** 2025-10-02

**Authors:** Anna Michaely, Hong Chen, Oliver Clemens, Maxim Neuberger, Christopher W. M. Kay, Robert Haberkorn, Guido Kickelbick

**Affiliations:** † Inorganic Solid-State Chemistry, 9379 Saarland University, Campus, Building C4.1, 66123 Saarbrücken, Germany; ‡ Institute for Materials Science, Materials Synthesis Group, 122279 University of Stuttgart, Heisenbergstraße 3, 70569 Stuttgart, Germany; § Physical Chemistry and Didactics of Chemistry, Saarland University, Campus, Building B2.2, 66123 Saarbrücken, Germany; ∥ London Centre for Nanotechnology, University College London, 17−19 Gordon Street, London WC1H 0AH, U.K.

## Abstract

Magnesium spinels such as MgAl_2_O_4_ and MgFe_2_O_4_ have been widely explored for energy storage and sensing applications, but MgV_2_O_4_ remains relatively unexplored despite its promising potential, e.g., as a battery electrode material. In this study, we report the first mechanochemical synthesis of MgV_2_O_4_ at room temperature using either MgO or Mg with various vanadium oxides as reactants. Directed by thermodynamic calculations on a DFT level, only the self-sustaining reaction between V_2_O_5_ and Mg led to MgV_2_O_4_ within 20 min of milling, along with MgO as a side product. With increasing rotational speed, an earlier reaction ignition after a few minutes of milling, smaller crystallite sizes in the nanometer range, and increased strain in MgV_2_O_4_ were observed. In addition, harsh milling conditions induce increasing nonstoichiometry in both phases, leading to a magnesium-rich spinel and a vanadium-containing rock salt phase, as supported by X-ray diffraction and electron paramagnetic resonance measurements. Acid washing after synthesis removed MgO, and electrochemical impedance spectroscopy showed that milder grinding conditions increased the conductivity of MgV_2_O_4_ due to the smaller number of defects.

## Graphical abstract

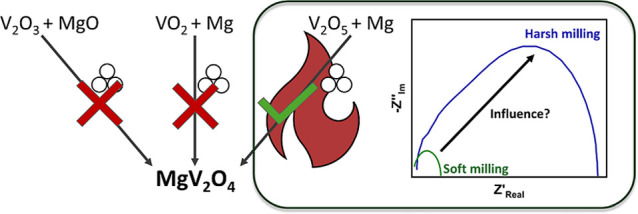

## Introduction

With over 200 literature known compounds and minerals,^1^ spinel compounds with the general sum formula AB_2_X_4_ (A, B = metal cation; X = O^2–^, S^2–^, Se^2–^, Te^2–^) represent an important class of materials. In the case of direct spinels with space group *Fd*3̅*m*, the crystal structure features a robust cubic closed packed substructure of oxygen atoms with 1/8 of the resulting tetrahedral sites being occupied by A cations and half of the octahedral sites by B cations.^2^ Due to their versatile elemental composition facilitated by a quite flexible cationic arrangement and incorporation of various transition metals, the resulting (redox)­chemical, electronic, and magnetic properties can be tailored for use in various applications such as catalysis,^3^ energy storage and batteries,^4^ sensors,^5^ or for oxygen reduction/evolution reactions (ORR/OER).^6^ For example, while MgAl_2_O_4_, which is one of the most studied spinels, is widely used in optical applications due to its transparency and high thermal stability;^7^ the magnetic properties of iron and the biocompatibility of magnesium make MgFe_2_O_4_ an ideal candidate for cancer treatment and other biomedical applications.^8^ Typical synthesis routes for the preparation of spinels include solid-state high temperature reactions starting from the corresponding oxides as reactants,^9^ thermal decomposition,^8a,10^ sol–gel processes,^11^ hydrothermal syntheses,^12^ microemulsion,^13^ and chemical vapor deposition.^14^

Mechanochemistry offers a greener, solvent-free, room-temperature alternative to conventional high-temperature or hydrothermal synthesis methods, which are often energy- and solvent-intensive.^15^ Depending on the type of ball mill and the milling intensity, ball milling can generally be classified into two types: low-energy and high-energy ball milling.^10^ Low-energy milling provides only a small energy input, is commonly carried out in tumbler mills, and primarily enables a homogeneous and uniform reduction of particle size.^16^ In some cases, sequential ball milling, meaning that the powder is subjected to multiple milling steps, has been applied successfully. For example, Farhad et al. were able to produce CuBi_2_O_4_ nanopowder with a tunable band gap for use in (photo)­electrochemical applications by using this approach.^17^ In contrast, high-energy milling not only reduces particle sizes but can also introduce defects and increase product reactivity due to the much larger energy input. This approach is also typically used when the induction of a chemical reaction in the ball mill is envisioned, as it can shorten reaction times, improve yields, or even enable product formation unattainable by conventional synthesis routes.^15,18^

While the mechanochemical synthesis and behavior of MgV_2_O_4_ remain largely unexplored, related spinels such as MgFe_2_O_4_ and MgAl_2_O_4_ have been synthesized using various precursors, including metal hydroxides,^19^ oxides,^19c,19d,20^ or a combination of both.^19c,19d,21^ Compared to more traditional approaches, mechanochemical routes can lead to enhanced magnetic^19a^ and catalytic properties of spinels.^20e^ However, a sintering step is often required to obtain phase-pure spinels.

Compared with other magnesium-based spinels such as MgAl_2_O_4_ and MgFe_2_O_4_, MgV_2_O_4_ has received relatively limited attention. The reported synthesis methods are primarily hydrothermal techniques^22^ or high-temperature solid-state reactions,^23^ while to our knowledge, no mechanochemical synthesis has been described to date. Despite the sparse literature, MgV_2_O_4_ is a promising electrode material for magnesium-ion^22a,22b,23d,24^ or zinc-ion batteries.^22c^ For example, Ding et al. demonstrated good cycling stability with a capacity of 112 mAh g^–1^ using cactus-like MgV_2_O_4_ structures as cathodes for Mg-ion batteries,^22b^ while Tang et al. reported a capacity of 272 mAh g^–1^ at 0.2 A g^–1^ for urchin-like morphologies.^22c^ Additionally, MgV_2_O_4_ is a key intermediate in the reduction of V_2_O_5_ by Mg in a self-propagating reaction to prepare elemental vanadium, which is an important additive for high-performance steels and alloys.^25^

Yang and Cormick demonstrated that milling V_2_O_5_ with 5 eq. Mg, Al, or Ti in a Spex 8000 mixer mill leads to the rapid and highly exothermic formation of elemental vanadium via a mechanochemical reduction reaction.^26^ Depending on the size of the milling balls, a combustion event was detected after an activation period between 4 and 570 s of milling, which was reflected in a sudden increase in the vial temperature. This combustion event indicates the onset of a mechanochemically induced self-propagating reaction (MSR). Unlike traditional self-propagating high-temperature synthesis, MSRs occur at room temperature within a ball mill and are driven by mechanical energy. A key criterion for MSRs is a high adiabatic temperature *T*_*ad*_, typically exceeding 1800 K, which can be estimated by dividing the reaction energy by the specific heat capacity.^27^ Ideally, both parameters are known for the same temperature. Other reported MSRs involving V_2_O_5_ have been reported for the formation of alloys^28^ or carbides.^29^ Recently, we also showed that milling V_2_O_5_ with NaH induces similarly exothermic reactions, yielding various sodium vanadium oxides.^30^

In this study, we investigate the mechanochemical synthesis of MgV_2_O_4_ using different combinations of vanadium oxides and magnesium sources, namely, V_2_O_3_/MgO, 2VO_2_/Mg, V_2_O_5_+V_2_O_3_ (formally VO_2_)/2Mg, and V_2_O_5_/2Mg. The investigation is based on quantum chemical calculations to assess the thermochemistry of possible reactions. Additionally, the electrochemical properties of the as-synthesized MgV_2_O_4_ are studied by electrochemical impedance spectroscopy (EIS).

## Experimental Section

### Materials

V_2_O_5_ (abcr, Karlsruhe, Germany, 99.9%), VO_2_ (ChemPur, Karlsruhe, Germany, 99.5%), MgO (N50 leicht, Lehmann&Voss&Co, Hamburg, Germany), vanadium powder (Fisher Scientific, Kandel, Germany, 99.5%), and magnesium powder (Thermo Scientific, Dreieich, Germany, ≥99.8%) were used without further purification and stored in a glovebox under an argon atmosphere. All solids have been characterized by X-ray diffraction before use. Due to partial conversion of MgO into Mg­(OH)_2_, MgO was annealed at 600 °C for 48 h in air before use as a reactant.

### Synthetic Procedures

#### Preparation of V_2_O_3_ via Solid-State Reaction

Stoichiometric amounts of V_2_O_5_ (3 equiv) and V (4 equiv) were mixed, placed in a porcelain boat, and heated to 900 °C under argon with a heating rate of 200 °C/h. The temperature was held for 12 h, followed by an intermediate grinding step and subsequent heating cycles at 900 C for 12 h until the retention of phase-pure V_2_O_3_.

#### Mechanochemical Syntheses

All syntheses were performed under an argon atmosphere by using a glovebox.

Stoichiometric amounts of V_2_O_3_/MgO, 2VO_2_/Mg, V_2_O_5_ + V_2_O_3_/2Mg, or V_2_O_5_/2Mg for the synthesis of MgV_2_O_4_ were lightly mixed with a spatula before mechanochemical treatment in the planetary ball mill Pulverisette 7 Premium line (Fritsch, Idar-Oberstein, Germany). The batch size was set to 3.0 g each time. In most experiments, the rotational speed was set to 300 rpm and the milling time was set to 20 min. Experiments that differ from the standard ones, using more intense milling conditions, are mentioned below. WC milling jars with a volume of 45 mL and 90 WC milling balls with a diameter of 5 mm were used. WC milling equipment was chosen due to small cracks of ZrO_2_ milling equipment in preliminary experiments, which were most likely caused by the mechanochemical reaction of V_2_O_5_ and Mg.

To remove the side product MgO after the reaction of V_2_O_5_ and Mg, samples were treated according to a modified literature procedure.^31^ After overnight stirring in 0.4 M HCl, the samples were treated with 7.5 M HCl for 1.5 h at 40 °C followed by an additional 0.5 h at room temperature. Subsequently, the samples were washed with distilled water until pH neutrality was reached and dried in a vacuum oven at 80 °C.

### Characterization

Powder X-ray diffraction (PXRD) patterns of the pulverized samples were recorded at room temperature on a D8-A25-Advance diffractometer (Bruker, Karlsruhe, Germany) in Bragg–Brentano θ-θ-geometry (goniometer radius of 280 mm) with Cu K_α_-radiation (λ = 154.0596 pm). A 12 μm Ni foil working as the *K*_β_ filter and a variable divergence slit were mounted at the primary beam side. A LYNXEYE detector with 192 channels was used at the secondary beam side. Experiments were carried out in a 2θ range of 7 to 120° with a step size of 0.013° and a total scan time of 2 h. Rietveld refinements of the recorded diffraction patterns were performed using TOPAS 5.0 (Bruker AXS, Karlsruhe, Germany) software.^32^ Crystallographic structure and microstructure were refined. The mean crystallite size <L> was calculated as the mean volume-weighted column height derived from the integral breadth. Since the width of reflections is influenced by both the microstructure of the sample and the instrumental setup, the fundamental parameters approach^33^ (implemented in Topas 5.0) was used to account for the contributions of instrumental line broadening, which enables microstructural analysis. Crystal structure data for Rietveld refinement were obtained from the Pearson’s Crystal database.^34^

Electron paramagnetic resonance (EPR) spectroscopy was performed at a temperature of 5 K using an ELEXSYS E580 (Bruker, Ettlingen, Germany) spectrometer at X-band frequencies (9–10 GHz) with a modified^35^ ER4118X-MD5 resonator (Bruker, Ettlingen, Germany) with symmetric long holders. For temperature control, a closed cycle helium cryostat (Cryogenic CF VTC) was used. Data evaluation and simulation were performed in MATLAB^36^ using the EasySpin^37^ toolbox.

The elemental quantification was conducted via inductively coupled plasma mass spectrometry (ICP–MS) with a commercial ICP–MS system (8900 Triple Quad and SPS4 autosampler, Agilent, Santa Clara, USA). Stock solutions of single-element ICP–MS standards of Mg­(II) (1000 mg L^–1^ in 2–3% HNO_3_, Merck Certipur, Darmstadt, Germany), V­(V) (10 g L^–1^ in 5% HNO_3_, Alfa Aesar, Karlsruhe, Germany), W­(VI) (1 g L^–1^ in 4% HNO_3_, Carl Roth, Karlsruhe, Germany), Ho­(III) (1 g L^–1^ in 2–3% HNO_3_, Merck Certipur, Darmstadt, Germany), and Sc­(III) (1 g L^–1^ in 5% HNO_3_, Alfa Aesar, Karlsruhe, Germany) were used. The last two are used as internal standards. The detector dwell time was 100 μs, the repetition was 3 times, and the measured isotopes were ^24^Mg, ^51^V, and ^182^W. For the ICP–MS measurements, approximately 3 mg of the sample was dissolved in 4 mL of aqua regia (HNO_3_ ROTIPURAN Supra 69%, Carl Roth, Karlsruhe, Germany; HCl Suprapur 30%, Merck, Darmstadt, Germany) before being diluted with ultrapure water. An external calibration was performed for quantification.

EIS measurements were carried under an air atmosphere using an MTZ-35 impedance analyzer (BioLogic, Seyssinet-Pariset, France) applying an AC signal of 100 mV amplitude (linearity of EIS data was confirmed and is demonstrated in Figure S1), with a frequency range from 1 MHz to 100 mHz. ∼200 mg of the powdery sample was pressed into pellets with a diameter of 7.3 mm by applying uniaxial pressure (2 t load) followed by isostatic pressing (400 kN). Isostatic pressing results in good stability of the pellet without using additional binder materials, which can impact the resistance to charge transfer between the grains. All samples were investigated over a temperature range of 298–373 K during the heating sweep and 373–273 K during the cooling sweep. The top and bottom of the pellets were sputtered with gold to establish the electrical contact to the electrodes of the spectrometer. The data were fitted using the software RelaxIS3 (rhd instruments, Darmstadt, Germany).

### Quantum Mechanical Calculations

Calculation of the free energy of the (magnesium) vanadium oxides at 0 K was carried out using the projector-augmented wave method by Blöchl^38^ implemented in the Vienna ab Initio Simulation Package (VASP) version 6.4.^39^ Exchange and correlation effects were accommodated using the General Gradient Approximation functional by Perdew–Burke–Ernzerhof optimized for bulk solids (PBEsol).^40^ POTPAW_PBE_54 potentials were used for all calculations, more specifically Mg_sv, V_sv, and O_h. The free energy of the spinel phase as well as that of reduced vanadium oxides was obtained with spin polarization. The convergence criteria for electronic and ionic relaxation were set to 10^–4^ and 10^–5^ eV, respectively, while the cutoff energy was set to 800 eV. The Brillouin zone was sampled by automatically generating a Γ-centered *k*-point mesh with a resolution of minimum 0.035 Å^–1^ per cell. For ease of comparison, the reaction enthalpies calculated from the respective free energies of the oxides are normalized to the atomic mass units (u) of the reactants.

## Results and Discussion

### Quantum Mechanical Calculations

Apart from hydrothermal and carbothermal synthesis, the successful synthesis of (nearly) phase-pure MgV_2_O_4_ has frequently been reported using V_2_O_3_ and MgO as starting materials.^23a–23c^ Given the various stable vanadium oxides, different combinations of magnesium/magnesium oxide and vanadium oxides (V_2_O_5_, VO_2_, V_2_O_3_) as reactants are possible. While combinations such as V_2_O_3_/MgO, 2VO_2_/Mg, or V_2_O_5_ + V_2_O_3_ (formally VO_2_)/2Mg would lead to pure MgV_2_O_4_, the combination of V_2_O_5_/2Mg would result in the formation of MgV_2_O_4_ and MgO as side products.

To evaluate the thermodynamic feasibility of the proposed reactant combinations and identify those most likely to proceed under mechanochemical conditions in a planetary ball mill, quantum mechanical calculations were conducted by using the Vienna Ab initio Simulation Package (VASP). As summarized in Table 1, the reaction between V_2_O_3_ and MgO is the only endothermic process with a reaction enthalpy of +0.34 eV. This endothermicity is attributed to the high thermodynamic stability of MgO, suggesting that this reaction is unlikely to be initiated under ball milling conditions. In contrast, all other reactant combinations are predicted to be exothermic, with reaction enthalpies ranging from −13.30 meV/u (VO_2_/Mg) to −27.89 meV/u (V_2_O_5_/Mg), indicating a probability of successful initiation during ball milling. These theoretical findings are further supported by trends derived from standard enthalpies of formation reported in the literature,^41^ which show consistent relative reaction enthalpies among the different reactant pairs. This agreement reinforces the reliability of computational predictions and highlights the potential of specific combinations for mechanochemical synthesis.

**Table 1:** Reaction Enthalpies Per mol MgV_2_O_4_ (Δ*H*_r_) and Reaction Enthalpies Per Atomic Mass Unit of the Reactants (Δ*H*_r,Reactant_) for the Formation of MgV_2_O_4_ with Different Vanadium and Magnesium Sources^a^

Nr.	reaction equation	Δ*H*_r_ (eV)	Δ*H*_r,reactant_ (meV/u)	Δ*H*_literature_ (eV)
1	V_2_O_3_ + MgO → MgV_2_O_4_	0.34	1.77	–0.20
2	2 VO_2_ + Mg → MgV_2_O_4_	–2.53	–13.30	–4.31
3	0.5 V_2_O_5_ + 0.5 V_2_O_3_ + Mg → MgV_2_O_4_	–3.05	–16.01	–4.72
4	V_2_O_5_ + 2 Mg → MgV_2_O_4_ + MgO	–6.43	–27.89	–9.23

a For comparison, the reaction enthalpies (Δ*H*_literature_) calculated from the standard enthalpies of formation tabulated in literature^41^ are given.

### Reaction of Vanadium­(III) Oxide and Magnesium Oxide

The feasibility of synthesizing MgV_2_O_4_ via mechanochemical activation of V_2_O_3_ and MgO was examined by subjecting the oxide mixture to ball milling at 300 rpm for up to 12 h, followed by an intensified milling step at 700 rpm for 1 h. Based on the calculated endothermic reaction enthalpy (Table 1), the formation of MgV_2_O_4_ under these conditions was considered thermodynamically unfavorable. PXRD measurements performed after 4 and 12 h of milling confirmed the persistent presence of both starting materials V_2_O_3_ (*R*3̅*c*, main reflections at ∼24.3, 33.0, 36.1, and 53.9° 2θ) and MgO (*Fm*3̅*m*, main reflections at ∼36.9, 42.9, and 62.3° 2θ) even after prolonged milling (Figure 1a). Although reflection broadening was observed, indicative of reduced crystallite size due to the continuous impact of the milling balls, no new reflections corresponding to MgV_2_O_4_ (*Fd*3̅*m*, main reflections at ∼36.9°, 42.9°, and 62.3° 2θ) were detected. Even after the additional milling step at 700 rpm for 1 h, the diffraction patterns remained unchanged, confirming the absence of product formation. These experimental findings are consistent with the theoretical prediction of an endothermic reaction pathway and underscore the utility of theoretical calculations in prescreening reactant combinations for mechanochemical synthesis. The inability to induce the reaction under the applied milling conditions highlights the importance of thermodynamic driving forces in successful mechanochemical transformations.

**Figure 1:**
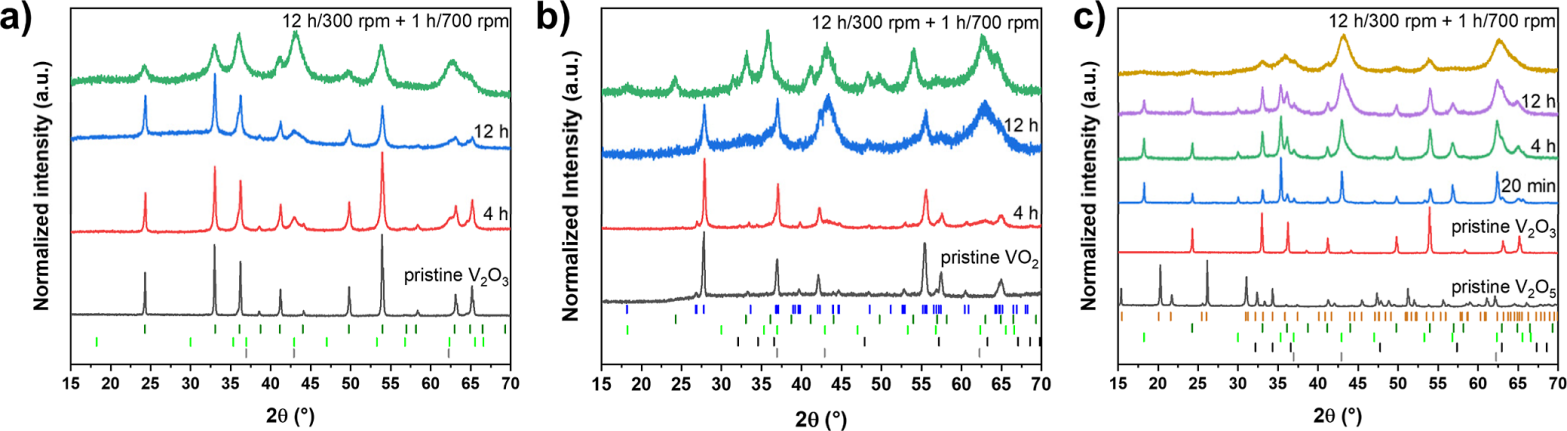
Normalized PXRD patterns after milling (a) an equimolar mixture of V_2_O_3_ and MgO, (b) VO_2_ and 0.5 eq. Mg, and (c) a mixture of V_2_O_5_, V_2_O_3_, and Mg. The respective milling times are given in each graph as well as the PXRD patterns of the corresponding pristine vanadium oxides. Unless otherwise indicated, the milling speed was set to 300 rpm. Bragg reflection positions are marked as follows: ocher ticks for V_2_O_5_ (*Pmmn*), blue for VO_2_ (*P*6_3_/*mmc*), dark green forV_2_O_3_ (*R*3̅*c*), light green for MgV_2_O_4_ (*Fd*3̅*m*), black for Mg (*P*6_3_/*mmc*), and gray for MgO (*Fm*3̅*m*).

### Reaction of Vanadium­(IV) Oxide and Elemental Magnesium

To further explore the mechanochemical synthesis of MgV_2_O_4_, a mixture of VO_2_ and 0.5 eq. of magnesium powder was subjected to the same milling protocol as previously applied to V_2_O_3_ and MgO. PXRD measurements revealed that milling at 300 rpm for up to 12 h did not result in a significant reaction progress. The diffraction pattern remained dominated by reflections corresponding to unreacted VO_2_ (*P*2_1_/*c*), with the main reflections observed at 27.8°, 36.9°, and 55.4° 2θ (Figure 1b). Subsequent milling at 700 rpm for 1 h did not result in the anticipated mechanochemical synthesis of phase-pure MgV_2_O_4_. However, a broad reflection at ∼18.3° 2θ suggests the possible formation of a minor fraction of highly nanocrystalline MgV_2_O_4_. More prominently, reflections corresponding to V_2_O_3_ (∼24.3°, 33.0°, 36.1°, and 53.9° 2θ) and MgO (∼36.9°, 42.9°, and 62.3° 2θ) were clearly detected, indicating that harsh ball milling of VO_2_ and Mg leads to the formation of V_2_O_3_ and MgO. This observation is consistent with DFT calculations, which show that the reaction enthalpy for the formation of V_2_O_3_ and MgO (−2.87 eV) is more exothermic than that for the formation of MgV_2_O_4_ (−2.53 eV). The thermodynamic preference for V_2_O_3_ and MgO instead of MgV_2_O_4_ under harsh milling conditions explains their predominance in the PXRD pattern. The relatively small difference in reaction enthalpies also accounts for the minor formation of MgV_2_O_4_, likely as a secondary product.

### Reaction of Vanadium­(III) Oxide, Vanadium­(V) Oxide, and Elemental Magnesium

Milling an equimolar mixture of V_2_O_3_ and V_2_O_5_ (formally VO_2_) with Mg, as described by reaction eq 3 (Table 1) resulted in a noticeable temperature rise in the milling jar after 20 min at 300 rpm, indicating the onset of an exothermic reaction. Upon opening of the milling jar, a fine black powder was obtained, and the characteristic orange color of V_2_O_5_ was no longer visible. PXRD analysis revealed the complete disappearance of reflections corresponding to V_2_O_5_ (*Pmmn*) and Mg (*P*6_3_/*mmc*) after just 20 min of milling, while reflections of unreacted V_2_O_3_ remain detectable even after 12 h (Figure 1c). Concurrently, new reflections attributed to MgV_2_O_4_ and MgO emerged and progressively broadened with an extended milling time and with continuous mechanical impact. The fact that only unreacted V_2_O_3_ was detected among the three reactants after 20 min of milling indicates that only the highly exothermic reaction between V_2_O_5_ and Mg proceeded. The activation energy required for the subsequent endothermic reaction between V_2_O_3_ and in situ formed MgO (Table 1) is again too high for induction in a ball mill.

### Reaction of Vanadium­(V) Oxide and Elemental Magnesium

#### Influence of the Ball Milling Procedure on the Sample Composition

Building on the promising results for the rapid mechanochemical synthesis of MgV_2_O_4_ with MgO as a byproduct from V_2_O_5_ and Mg, pure V_2_O_5_ and Mg were milled together at 300 rpm. No significant temperature increase in the milling jar was observed after 5 min. However, a noticeable rise in jar temperature occurred between 5 and 10 min of mechanochemical treatment, indicating the onset of an exothermic reaction and a black powder accompanied by grains of molten material were obtained. Extending the milling time to 20 min ultimately resulted in a fine black powder. The presence of molten grains and the observed temperature spike suggest a combustion event characteristic of a self-propagating reaction. This behavior aligns with our previous studies of mechanochemical self-propagating reactions between V_2_O_5_ and NaH,^30^ the high exothermicity reported for the vanadium formation from V_2_O_5_ and Mg^26^ and predictions from our VASP calculations. Due to prior damage to sensor and milling equipment after milling V_2_O_5_ with Mg, the recording of an in situ pressure and temperature curve with the EASY GTM system by Fritsch to determine the reaction onset was not performed in this case.

Increasing the rotational speed to 500 and 700 rpm leads to earlier reaction initiation, with the milling jar feeling warm after just 5 min of mechanochemical treatment in both cases.

PXRD measurements confirm that MgV_2_O_4_ is successfully formed with all investigated rotational speeds, with MgO consistently appearing as a byproduct (Figure 2a). With increasing milling time, reflections progressively broaden, which indicates a decrease in the crystallite size. Rietveld refinements using both stoichiometric MgV_2_O_4_ and MgO phases reveal a steady increase in the MgO content, rising from 19 wt% at 300 rpm to 61 wt% at 700 rpm (Figure 2b). This trend suggests a compositional shift in the overall sample, evolving from Mg_2.10_V_2_O_5.10_ at 300 rpm, which closely matches the theoretical composition of Mg_2_V_2_O_5_ based on the initial reactant masses, to Mg_8.25_V_2_O_11.25_ at 700 rpm, which implies an unrealistic vanadium deficiency in the sample.

**Figure 2:**
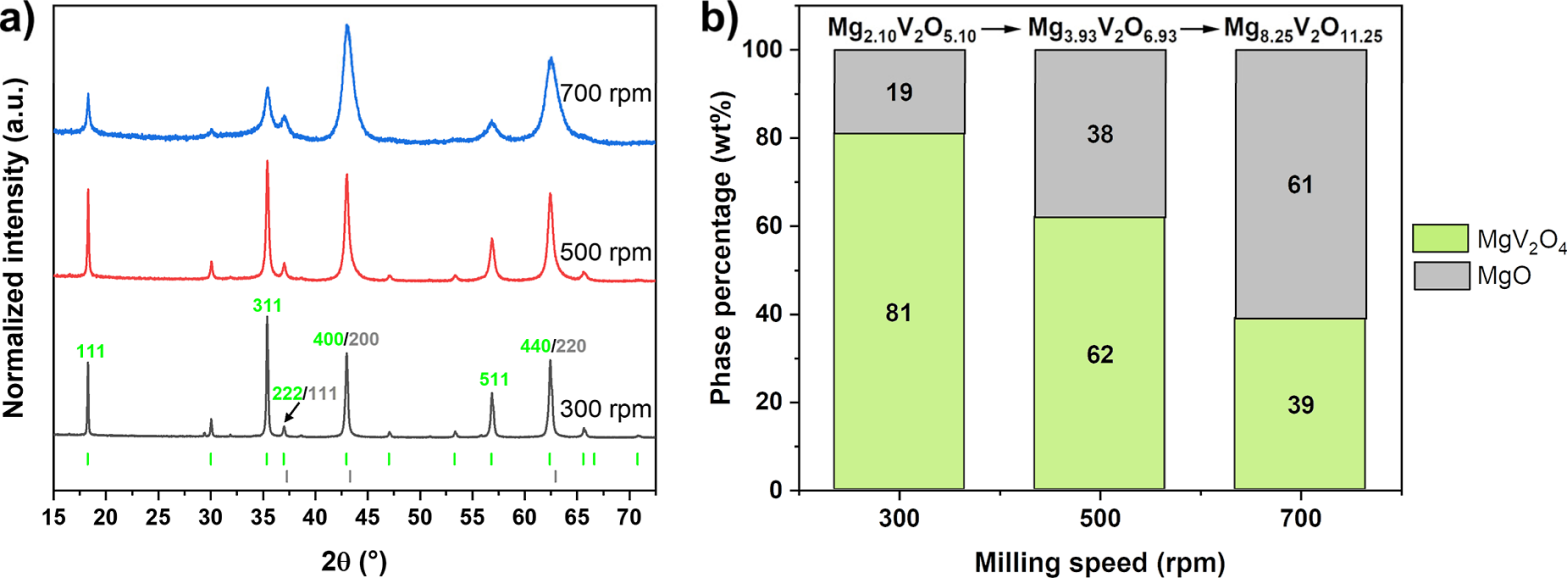
(a) Normalized PXRD diffractograms of MgV_2_O_4_ prepared mechanochemically at 300 (black), 500 rpm (red), and 700 rpm (blue) in 20 min. Bragg reflection positions are indicated by green ticks for MgV_2_O_4_ (*Fd*3̅*m*) and gray ticks for MgO (*Fm*3̅*m*). Indexing of selected reflections of MgV_2_O_4_ is given in green and of MgO in gray. (b) Corresponding evolution of the sample composition as a function of the milling speed as determined by Rietveld refinement, assuming stoichiometric compounds.

In theory, this apparent deficiency might be explained by the presence of an amorphous or highly nanocrystalline phase with an unknown composition. Quantitative analysis, including the use of an internal standard, was employed to estimate the amorphous content (see the Supporting Information for further details). The results indicate that samples milled at lower rotational speeds (300 rpm) contain no or only minimal amounts of the amorphous phase (maximum 5%), whereas samples subjected to more intense milling conditions (700 rpm) exhibit an increased amorphous fraction of 12–15%. While this trend aligns with expectations for ball-milled materials, the overall amorphous content remains moderate.

However, a thorough examination of the MgO phase after milling V_2_O_5_ and 2 eq. Mg for 20 min further reveals a shift in the relative intensities of reflections (111) at 36.9° 2θ and (200) at 42.9° 2θ, indicating that the cationic positions scatter more than expected for pure MgO. Furthermore, the lattice parameter of MgO decreases from 421.47(1) pm at 300 rpm, consistent with literature,^42^ to 419.8(3) pm at 700 rpm, which is smaller than expected. Given that VO (*Fm*3̅*m*) exhibits a lattice parameter of 409.5 pm,^43^ this contraction could imply a partial vanadium incorporation into the MgO lattice under harsh milling conditions, resulting in nonstoichiometry in MgO.

In contrast, the lattice parameter of the spinel phase shows only a slight increase from 841.11 to 841.91 pm, remaining largely consistent with reported values.^44^ Assuming a nonstoichiometric, Mg-rich spinel, an increase in cell volume would be expected due to the larger cationic size of Mg^2+^ (cationic radius in octahedral geometry: 72.0 pm^45^) compared to V^3+^ (64.0 pm). However, to maintain charge neutrality, the substitution of two V^3+^ ions by one Mg^2+^ ion and one V^4+^ ion (58.0 pm) would counterbalance the expected expansion. This compensation likely accounts for the relatively stable lattice parameter and supports the hypothesis of mechanochemically induced nonstoichiometry in the spinel phase.

To account for the observed compositional variations, several mechanistic scenarios involving nonstoichiometric product phases can be proposed, each dependent on the milling intensity. These are summarized by the following reaction pathways:

1. Formation of stoichiometric MgV_2_O_4_ and MgO at low milling intensities:

1 $$V_{2}O_{5}+2Mg\to {MgV}_{2}O_{4}+MgO$$

2. Formation of stoichiometric MgV_2_O_4_ and insertion of V­(III) into MgO at intermediate milling intensities:

2 $$V_{2}O_{5}+2Mg\to \frac{1-5x}{1-4x}MgV_{2}O_{4}+\frac{1-3x}{1-4x}{Mg}_{1-3x}V_{2x}O$$

3. Formation of nonstoichiometric MgV_2_O_4_ and insertion of V­(II) into MgO at high milling intensities:

3 $$V_{2}O_{5}+2Mg\to {Mg}_{1+x}{V_{2-}}_{x}O_{4}+{Mg}_{1-x}V_{x}O$$

While the above postulated reaction pathways mainly serve as models to better understand the processes during milling, it is most likely that a combination of these reactions occurs simultaneously. However, the share of a specific pathway in the total reaction differs, according to the milling conditions.

Rietveld refinements based on these scenarios reveal that the choice of the structural model has only a minor impact on refined lattice parameters and microstructural features. However, for samples subjected to more intense milling, allowing for nonstoichiometry slightly improves the fit quality. Importantly no evidence for antisite disorder was detected in the MgV_2_O_4_ spinel phase.

For the sample milled at 300 rpm, refinement yields a phase composition of 81% MgV_2_O_4_ and 19% MgO, corresponding to an overall sample composition of Mg_2.10_V_2_O_5.10_ (Supporting Information, Figure S2, and Table S1). Introducing nonstoichiometry into the refinement model does not significantly alter site occupancies, supporting the formation of stoichiometric MgV_2_O_4_ and MgO under mild milling conditions.

Independent of the different scenarios postulated above, a sample composition of 62% spinel and 38% rock salt was refined for the 500 rpm sample (Supporting Information, Figure S3, and Table S2). While the spinel phase remains stoichiometric, up to 26% of vanadium can be incorporated into the MgO lattice, depending on the assumed oxidation state, leading to slightly increased quality fit parameters. Hereby, the refined overall sample composition of Mg_1.94_V_2_O_4.94_ for V­(III) according to scenario 2 is in excellent agreement with the theoretical one. While it can be approximated that mainly V­(III) is incorporated into the MgO lattice under intermediately harsh milling conditions, it is not possible to distinguish between two oxidation states by Rietveld refinement. Given that the rock-salt-type phase is considered a byproduct and will be removed in subsequent processing steps to isolate the spinel, no further structural characterization of this phase was pursued.

Allowing for nonstoichiometry in MgV_2_O_4_ and MgO and vanadium incorporation into the MgO lattice leads to a shift in the refined phase composition by up to 6% for the sample milled at 700 rpm, compared to the model assuming fully stoichiometric phases (Supporting Information, Table S3). Although the visual appearance of the fits remains largely unchanged across the different models (Supporting Information and Figure S4), the refinement quality indicators improve, and the overall sample compositions approach the theoretical composition. An approximate composition of Mg_1.5_V_1.5_O_4_ and Mg_0.77_V_0.23_O was refined for the spinel and rock salt structure, respectively.

These findings support the previously proposed mechanistic scenarios and indicate that magnesium-rich spinels can be accessed under high-energy milling conditions. However, further intensification of the milling process at 700 rpm revealed a significant decline in the structural integrity of the spinel phase. This is evidenced by the near-complete disappearance of spinel reflections in the PXRD pattern (Supporting Information, Figure S5), suggesting low mechanochemical stability under such conditions.

Consequently, while harsh milling may facilitate the formation of nonstoichiometric spinels, it is not suitable for the targeted synthesis of stoichiometric and well-crystalline MgV_2_O_4_. Controlled milling conditions are therefore essential to balance phase formation with structural preservation.

#### Analysis of Mechanochemically Induced Structural Changes by EPR Spectroscopy

EPR spectroscopy was employed to further investigate the structural modifications induced by mechanochemical treatment, with a particular focus on the local environment of vanadium species. In general, it would be expected that only the paramagnetic V^4+^ ion with its 3d^1^ electronic configuration is observed in the EPR spectra. Lower oxidation states such as V^3+^ and V^2+^ are unlikely to be observable at X-band frequencies due to strong fine structure interactions in the 3d^2^ and 3d^3^ configurations.^46^ The sensitivity of EPR to local structural distortions makes it therefore a valuable tool for detecting defects introduced during the mechanochemical reaction between Mg and V_2_O_5_. All recorded spectra exhibit a well-resolved hyperfine structure arising from the coupling of the paramagnetic species with the ^51^V nucleus (I = 7/2 and natural abundance of 99.75%). Additionally, a broad underlying resonance is observed, attributed to spin–spin exchange interactions between neighboring vanadium centers, consistent with previous reports on vanadium-containing systems.^47^ In accordance with the PXRD results, the EPR spectra show a strong influence of the milling intensity on the vanadium environment in the sample, as the appearance of the EPR signal continuously changes as a function of the milling intensity (Figure 3a).

**Figure 3:**
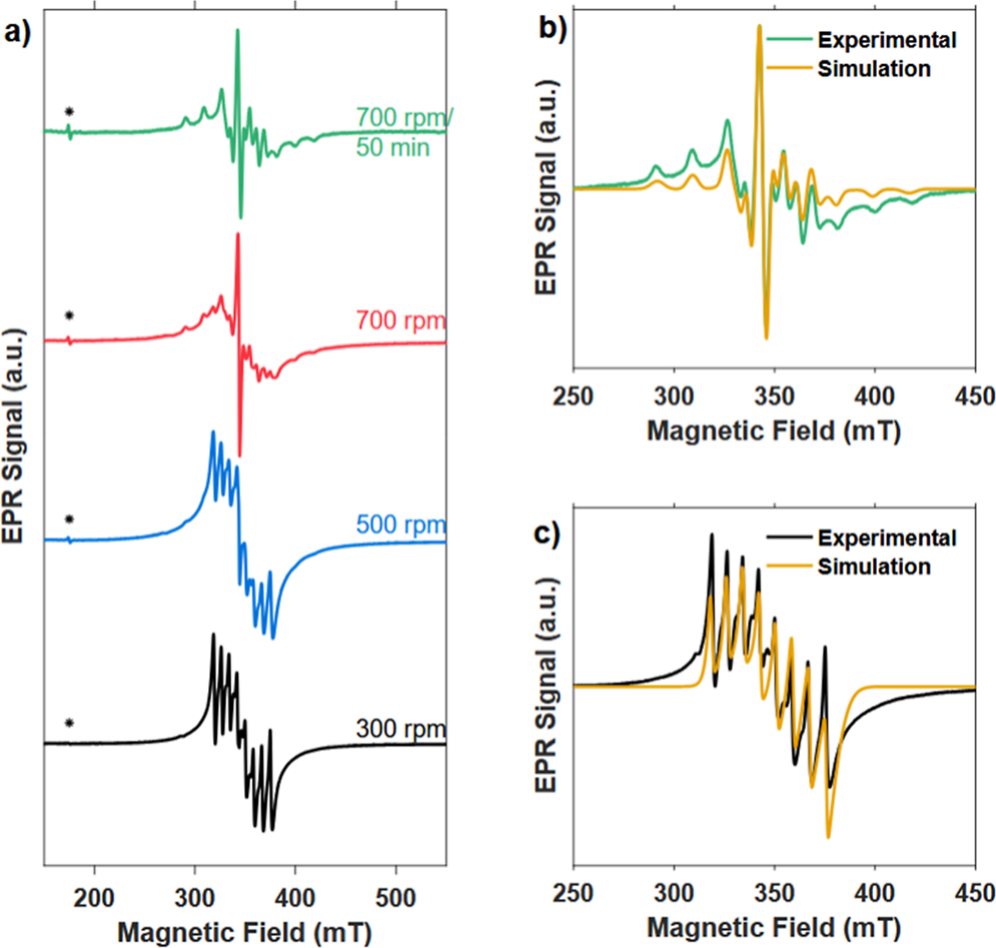
(a) Overview of continuous wave EPR spectra at 5 K of MgV_2_O_4_ prepared in 20 min at 300 (black), 500 (blue), and 700 rpm (red), and at 700 rpm in a total of 50 min of milling (green). The asterisks mark the signal from the microwave resonator. Experimental and simulated spectra of MgV_2_O_4_ prepared at 300 rpm in 20 min and at 700 rpm in 50 min are shown in (b) and (c), respectively.

Milling at 700 rpm for a total time of 50 min leads to a clear axial hyperfine tensor for coupling between the unpaired electron and the ^51^V nucleus in an octahedral environment. Simulation of the obtained spectrum (Figure 3b) reveals hyperfine tensor components of *A*_⊥_ = 158(1) MHz as well as *A*_∥_ = 495(3) MHz, which align well with those reported for similar vanadate systems.^47,48^ These results support the hypothesis of vanadium incorporation into the MgO matrix and the concurrent formation of a magnesium-rich spinel phase. At low and intermediate milling speeds of 300 and 500 rpm, the spectrum consists of eight resonance lines, which is expected for hyperfine coupling to the vanadium nucleus. The spectrum of the 300 rpm sample could be well simulated (Figure 3c) by assuming an axial g-tensor with *g*_⊥_ = 1.940(3), *g*_∥_ = 1.989(1) and an axial hyperfine tensor *A* with *A*_⊥_ = 120(3) MHz and *A*_∥_ = 227(1) MHz. More details of the simulation parameters are included in Supporting Information Tables S4 and S5. We note that a comparably large *g* strain had to be considered for an accurate simulation of the spectrum. Both the hyperfine coupling tensor and the significantly narrower lines of the EPR signal of the 300 rpm sample suggest a more isotropic environment of the paramagnetic centers, which is likely to arise from local defects generated during mechanochemical milling.

Although the exact nature of the paramagnetic centers remains uncertain, the significant changes in the EPR signal hyperfine coupling tensor after intense milling, suggesting the incorporation of vanadium into the crystal lattice, along with structural changes observed via Rietveld refinement, support the hypothesis that vanadium is incorporated into the MgO lattice under the formation of a defective rock salt structure and a magnesium-rich spinel. Again, intense milling is therefore not beneficial for the mechanochemical synthesis of MgV_2_O_4_.

#### Removal of MgO by Acid Washing

To remove the side product MgO, a modified literature procedure was employed involving sequential acid treatments: in 0.4 M HCl for 15 h, followed by treatment with 7.5 M HCl for 1.5 h at 40 °C and 0.5 h at room temperature. The samples were afterward washed with distilled water until neutral pH was achieved.^31^

After washing, reflections of (overlapping) MgV_2_O_4_ and MgO at ∼ 43° and 62° 2θ become noticeably narrower after washing (Figure 4a), indicating effective removal of MgO. However, because the main reflections completely overlap, it remains difficult to reliably quantify the remaining rock salt phase using Rietveld refinement. For samples milled at 300 and 500 rpm, Rietveld analysis suggests that less than 5% of a rock salt phase may be present, but equally good fits can be achieved without using MgO at all. In contrast, for the sample milled at 700 rpm, the use of MgO is still required for adequate refinement. Since the phase percentage of the rock salt phase decreased significantly from 55% to 21% after washing (Supporting Information, Table S7), it can be concluded that the majority of MgO was removed, which aligns with EPR and ICP–MS analysis. While EPR spectra of the 700 rpm sample still show the largest axial contribution after washing (Supporting Information, Figure S6), ICP–MS measurements reveal a decrease in the Mg to V ratio from approximately 1 to 1 in the unwashed samples to ∼0.6 to 1 after washing (Supporting Information, Table S8), showing that most of MgO is removed during acid washing.

**Figure 4:**
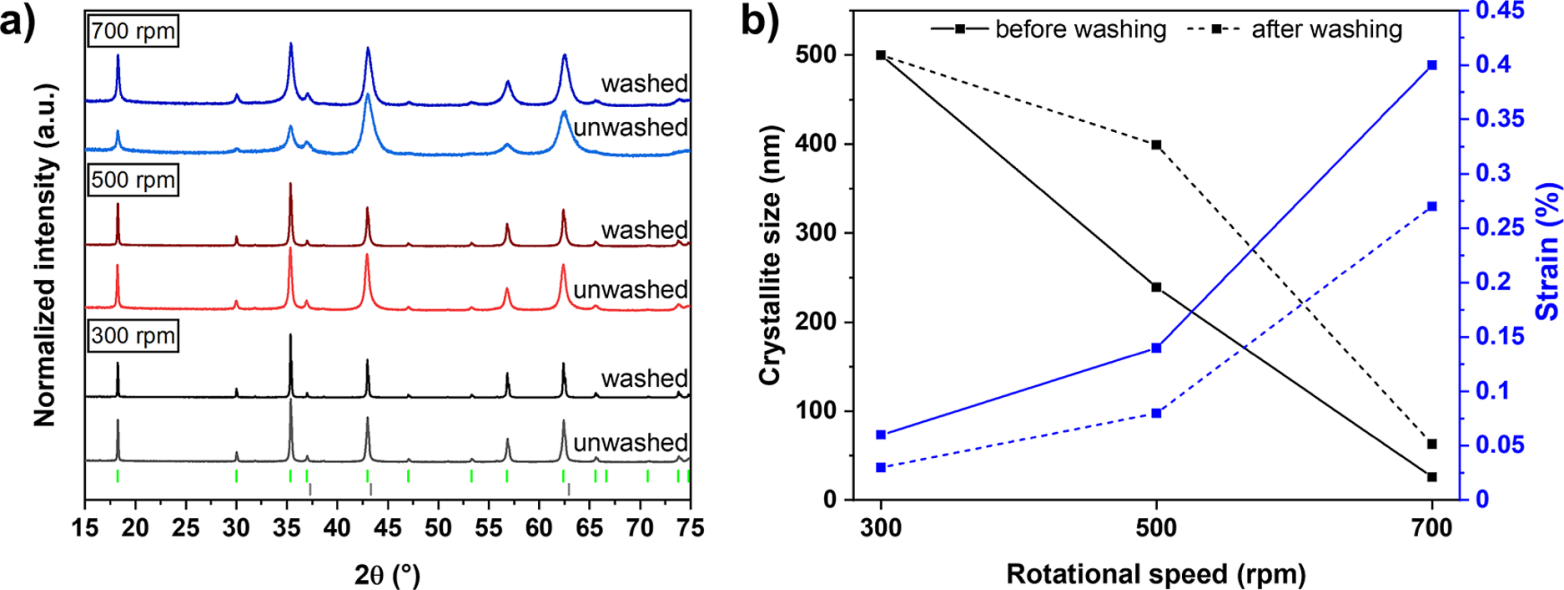
(a) Normalized PXRD diffractograms of MgV_2_O_4_ prepared mechanochemically before and after acid washing prepared at 300 (gray/black), 500 rpm (red), and 700 rpm (blue) in 20 min. Green ticks indicate the Bragg positions of MgV_2_O_4_ (*Fd*3̅*m*), gray ones of MgO (*Fm*3̅*m*). (b) Evolution of the crystallite size (black) and strain (blue) of the spinel phase as a function of the milling speed before (solid line) and after (dotted line) washing with diluted HCl. The lines in the figure connecting the points serve as orientation aids for the eye.

Regarding the microstructure of unwashed samples, the crystallite size decreases from >500 to 26 nm with intensifying milling conditions (Figure 4b), which is in line with a decrease of the particle size revealed by scanning electron microscopy (SEM) measurements (Supporting Information, Figure S7). Simultaneously, the strain of the spinel increases from 0.06 to 0.40% due to the harsher milling impact, which is a well-known phenomenon in mechanochemistry.^49^ After washing, an increase in crystallite size and a decrease in strain are observed in comparison to the unwashed samples. Rather than an improvement of the overall crystallinity due to crystal growth during washing, this observation is most likely caused by the dissolution of the smaller, more strained spinel crystallites in HCl as evidenced by a green coloration of the acid, which corresponds to dissolved V^3+^. These findings suggest that prolonged washing or the use of stronger acids are not advisable even though full removal of the side product might be possible due to the undesirable dissolution of the spinel phase.

#### Investigation of the Electrochemical Properties by Impedance Spectroscopy

To evaluate the impact of ball milling conditions on the electrochemical performance of the synthesized MgV_2_O_4_ samples, alternating current EIS was conducted on pellets prepared by isostatic pressing of nonsintered powders. This method allows for the assessment of total conductivity without the influence of sintering-induced microstructural changes. It is well established that high-energy ball milling reduces the particle size and increases the defect density, which can enhance the electrochemical activity. However, excessive milling may also introduce detrimental effects, such as impaired electron transport due to defect accumulation and grain boundary proliferation.^50^

The finer particle size resulting from high-energy milling increases the number of ceramic–ceramic interfaces in the pressed pellets, which can significantly influence both electronic and ionic transport. Although isostatic pressing improves pellet densification and mechanical integrity, samples still contain large amounts of grain boundaries in comparison to well-sintered ones.

As shown in the Nyquist plots of the MgV_2_O_4_ samples (Figure 5a–c), the shape of the semicircle as well as the frequency dependence of phase angles strongly depend on the milling speed. The absent blocking tail in all spectra in the low-frequency range indicates the electron conducting nature of the samples. Among the samples, the 300 rpm sample exhibits the lowest total resistivity over the temperature range from 0 to 100 °C. Increasing the milling speed from 300 to 700 rpm leads to a noticeable decrease of the conductivity from 52.2 to 8.59 μS/cm, respectively.

**Figure 5:**
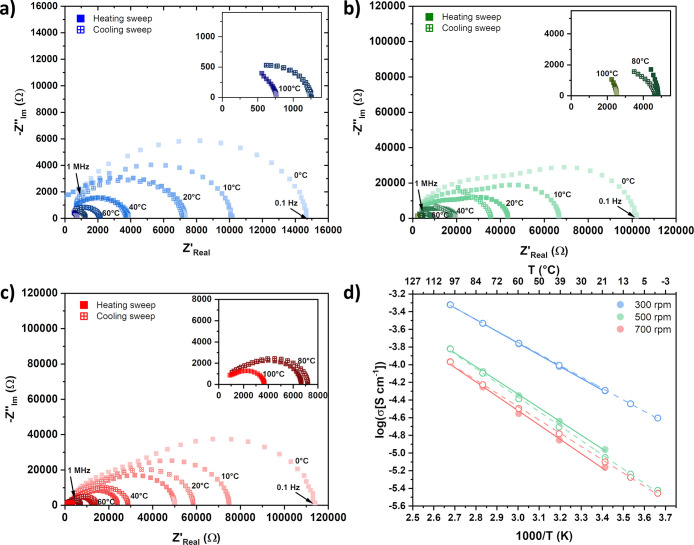
Nyquist plot of MgV_2_O_4_ prepared at (a) 300, (b) 500, and (c) 700 rpm as well as (d) the corresponding Arrhenius plot for all samples, separately for the heating (hollow circles) and cooling sweep (filled circles).

Arrhenius plots for all samples (Figure 5d) show excellent linearity (*R* ^2^ > 0.999), confirming thermally activated conduction behavior. Notably, the total conductivity decreases by approximately 1 order of magnitude with increasing milling speed. This trend correlates with the increased point defect density and grain boundaries introduced by the high-energy ball milling process, which is in line with the increase in strain and decrease in crystallite size as well as the nonstoichiometry observed by PXRD and EPR measurements. The accumulation of point defects and grain boundaries likely acts as scattering centers and barriers to charge transport, thereby reducing conductivity.^50,51^ This negative impact is also indicated by the higher activation energies for the 500 and 700 rpm samples (Table 2). Interestingly, the heating process affects the overall conductivity of the three samples differently. The 300 rpm sample shows negligible thermal hysteresis, while 500 and 700 rpm samples show either a slightly enhanced conductivity or a reduction of conductivity in a subsequent cooling sweep. The maximum deviation in conductivity reaches approximately 20%. Although the exact mechanism remains unclear, this behavior may be linked to milling-induced nonstoichiometry, defect dynamics, or moisture interactions at grain boundaries during thermal cycling in air.

**Table 2:** Calculated Activation Energy for All Samples, with Values Shown Separately for Heating and Cooling Cycles

milling speed (rpm)	measurement condition	*E*_a_ (eV)
300	heating sweep	0.26
	cooling sweep	0.26
500	heating sweep	0.32 (*E*_a,grain_: 0.28; *E*_a,gb_: 0.36)
	cooling sweep	0.31
700	heating sweep	0.30
	cooling sweep	0.32

To understand the rpm-dependent conductivity of different electron transport phenomena, the impedance data were evaluated by using equivalent circuit models consisting of R-CPE elements or series of them. The fitting examples for the three samples are shown in Figure 6a–c. As illustrated in the inset, *R* is a resistor and CPE is the constant phase element, which represents the nonideal capacitor response from samples due to surface roughness, porosity, and other microstructural imperfections of the pellet. The corresponding capacitances of CPE were determined using the Brug equation

$$C=Q^{1/\alpha }{\times R}^{1-\alpha /\alpha }$$

**Figure 6:**
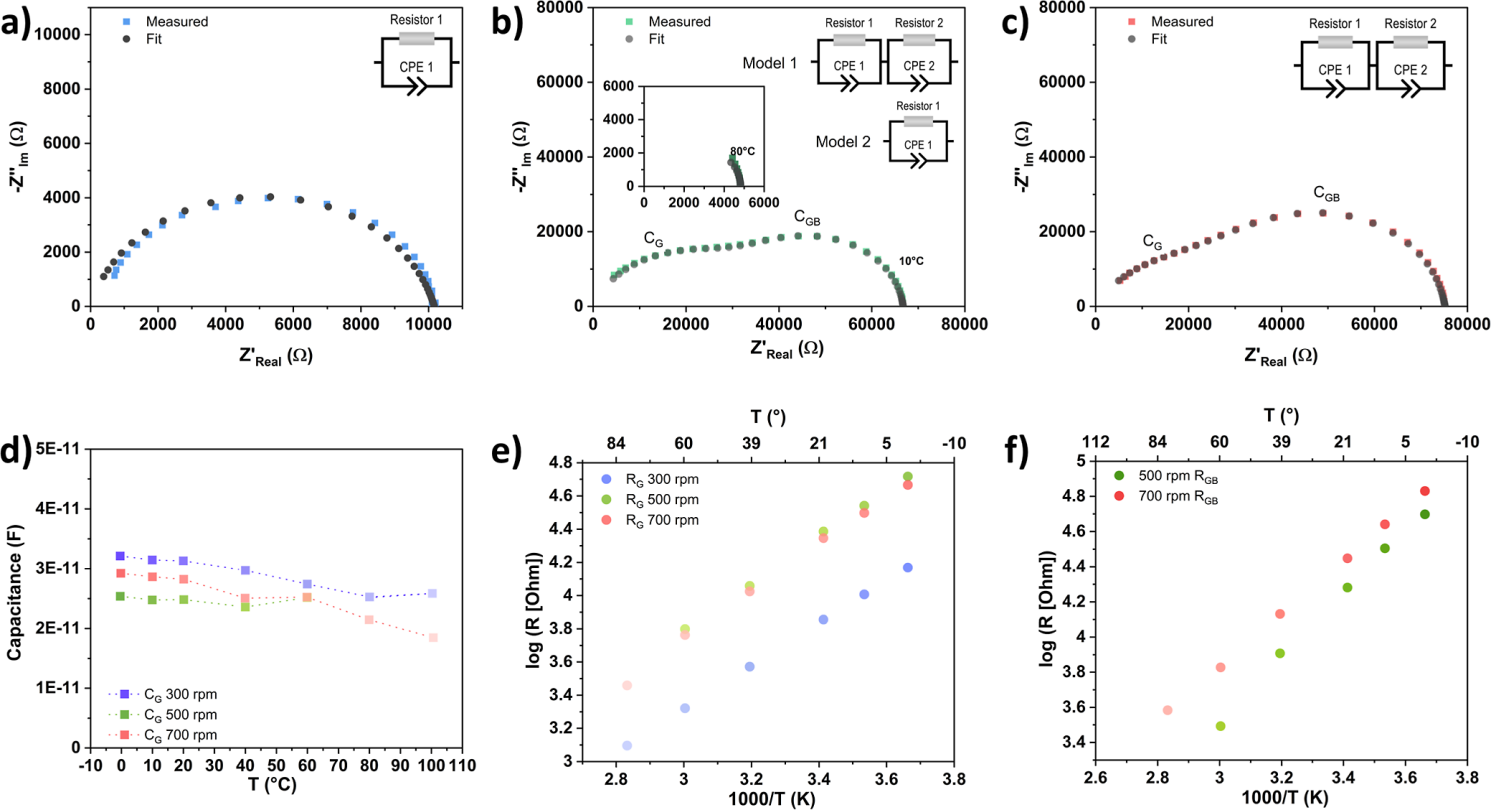
Fitting examples and models used for MgV_2_O_4_ prepared at (a) 300 rpm, (b) 500 rpm, (c) 700 rpm; (d) temperature-dependent bulk­(grain) capacitance for different samples; (e) temperature-dependent logarithm plot of bulk (grain) resistance and (f) grain boundary resistance for different samples.

*Q* is the CPE constant, *R* is the resistance in parallel with the CPE, and α is the CPE exponent (0 ≤ α ≤ 1). *Q*, *R*, and α can be extracted from the fitted spectra.

For the sample milled at 300 rpm, only a single R-CPE element was sufficient to model the data. Associated capacitance values in the range of 10^–11^ F were observed (Figure 6d), which exhibit a small dependence on temperature. For 500 and 700 rpm samples, equivalent circuits consisting of two single R-CPE elements were used (for the 500 rpm sample, at temperature above 80 °C, a single R-CPE element was used). The capacitance corresponding to the semicircle in the high-frequency range is also in the order of 10^–11^ F. Given the comparable temperature-dependence of the capacitances (Figure 6d), the associated resistance is attributed to the bulk transport.^52^ Figure 6e shows the grain resistance as a function of temperature (logarithmic scale) for the three samples during the heating sweep. A significant reduction in bulk conductivity by 1 order of magnitude, similar to the trend of overall conductivity, is observed. However, the 500 rpm sample shows marginal differences in bulk resistance to the 700 rpm sample, suggesting that the total conductivity differences among the samples are mainly due to nonbulk contributions.

For the 500 rpm sample (at temperatures below 80 °C) and 700 rpm sample, a second semicircle in the midfrequency range can be assigned to grain boundary contribution with capacitances in the range of ∼10^–10^ F. Figure S8 displays the grain, grain boundary, and total resistance as a function of temperature (logarithmic scale) for the 500 rpm sample. During the heating sweep, the electron-conducting phenomena are dominated by both bulk and grain boundary contributions at low temperatures, but by bulk resistance at high temperatures. This is consistent with the higher activation energy for grain boundary contribution as compared to the bulk contribution shown in Table 2. As illustrated in Figure 6f, the 700 rpm sample exhibits significantly increased grain boundary resistance (lower conductivity) compared to the 500 rpm sample, which is attributed to the increased density of grain boundary resulting from higher milling energy.^53^ The increased grain boundary resistance explains the lowest overall conductivity of the 700 rpm sample, especially given that the bulk resistance of this sample is comparable to that of the 500 rpm sample.

As the temperature increased, also during the subsequent cooling sweep, the 500 rpm sample exhibits only a single depressed and partial semicircle. Therefore, model 2 with only one R-CPE element was applied, as no indication of the presence of two semicircles was observed in the Bode plot either (Supporting Information, Figure S9). The corresponding capacitance was around 10^–10^ F. Despite this, it did not follow the tendency seen in model 2, neither for bulk resistance nor for grain boundary resistance (Supporting Information, Figure S8). This implies that the complexity of spectra and the mixed frequency domain of bulk and grain boundary contributions could not be fully deconvoluted with the current data set, and further investigations would be needed. However, a clear influence of the milling speed was observed by EIS measurements, revealing the positive influence of rather soft milling conditions, which further agrees with the observed low mechanochemical stability of the spinel.

## Conclusion

This study presents the first systematic investigation of the mechanochemical synthesis of MgV_2_O_4_ at room temperature, evaluating various combinations of vanadium oxides and Mg or MgO as precursors. Among the tested systems, only the highly exothermic reaction between V_2_O_5_ and Mg led to the successful formation of MgV_2_O_4_, accompanied by MgO as a side product, in agreement with the DFT calculations. In contrast, no reaction occurred between V_2_O_3_ and MgO due to the endothermic nature of the process, while reactions involving VO_2_/Mg and V_2_O_5_ + V_2_O_3_/Mg primarily resulted in partial reduction to V_2_O_3_ and selective reactivity of V_2_O_5_, respectively.

The self-propagating reaction between V_2_O_5_ and Mg was found to be sensitive to the milling intensity. Higher rotational speeds accelerated reaction initiation and led to reduced crystallite sizes and increased strain in the resulting MgV_2_O_4_. However, excessive milling introduced nonstoichiometry and structural instability, as evidenced by PXRD and EPR analyses, indicating that harsh milling conditions are detrimental to phase purity and structural integrity.

Following acid washing to remove MgO, the electrochemical properties of the as-prepared MgV_2_O_4_ pellets were evaluated by using EIS. While all samples showed capacitances of 3.0 ± 0.5 × 10^–11^ F, the results revealed that moderate milling conditions (e.g., 300 rpm) yielded the highest conductivity, while higher milling speeds led to increased resistivity. This decline in performance is attributed to the accumulation of structural defects and elevated grain boundary resistance, highlighting the importance of optimizing milling parameters to balance reactivity and material quality.

Overall, this work demonstrates the feasibility of synthesizing MgV_2_O_4_ via a rapid, room-temperature mechanochemical route and underscores the critical role of milling conditions in tailoring the structural and electrochemical properties of the resulting material for potential battery applications.

## Supplementary Material



## Notes

Additional powder X-ray and Rietveld refinement data as well as additional EPR, ICP–MS, SEM, and EIS data (PDF)
