# Supplementary material for: Mechanochemical Synthesis of MgV2O4: Reactivity Pathways Driven by Milling Energy and Precursors
Source: Inorg Chem. 2025 Oct 2;64(40):20163–74. doi: 10.1021/acs.inorgchem.5c03095 (PMC12522128; doi:10.1021/acs.inorgchem.5c03095)
Supplement: Supplementary file 1 [file ic5c03095_si_001.pdf]

Supporting Information

Mechanochemical Synthesis of MgV_2O_4: Reactivity Pathways Driven by Milling Energy and Precursors

Anna Michaely[1], Hong Chen[2], Oliver Clemens[2], Maxim Neuberger[3], Christopher W. M. Kay[3,4], Robert Haberkorn[1], Guido Kickelbick[1]*

[1] Saarland University, Inorganic Solid-State Chemistry, Campus, Building C4.1, 66123 Saarbrücken, Germany

[2] Institute for Materials Science, Materials Synthesis Group, University of Stuttgart, Heisenbergstraße 3, 70569 Stuttgart, Germany

[3] Physical Chemistry and Didactics of Chemistry, Saarland University, Campus, Building B2.2, 66123 Saarbrücken (Germany)

[4] University College London, London Centre for Nanotechnology, 17–19 Gordon Street, London WC1H 0AH (UK)

E-mail: guido.kickelbick@uni-saarland.de

1 Experimental Section – Testing of Linearity

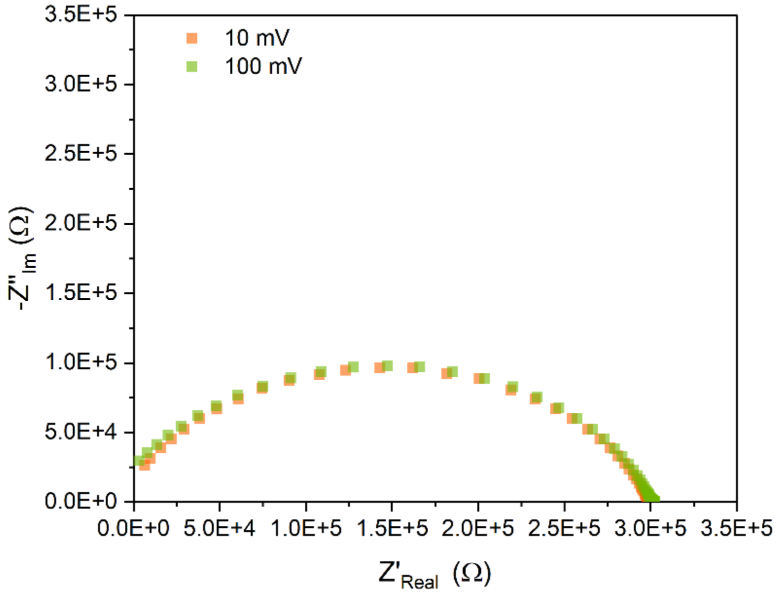

Figure S1: Nyquist plot of MgV_2O_4 sample measured with different amplitude values: 10 mV and 100 mV at 27 °C, respectively. The linearity of EIS data obtained by 100 mV in the manuscript is confirmed by the fact that no distorted data is observed between measurements using different perturbations.

2 Rietveld Refinement after ball milling V_2O_5 and Mg

2.1 Estimation of an amorphous phase

To estimate the phase percentage of an amorphous phase via Rietveld refinement, the well-established method of an internal standard can be used. After addition of a known weight percentage of a well crystalline, fine-grained compound such as CaF_2 (*wt% of spiked*), the percentage of amorphous phase in the sample (*wt% in original sample*) can be calculated via the following equation. Hereby, *wt% in spiked sample* represents the “absolute phase amount in the spiked sample after considering amorphous phase amounts” given in Topas 5.0.[1]

$$wt\%\ in\ original\ sample = \frac{wt\%\ in\ spiked\ sample}{1 - wt\%\ of\ spiked}$$

For harshly milled samples, estimation of the amorphous phase was also done via an interpretation of the background profile with a method related to PONKS.[2]

2.2 300 rpm

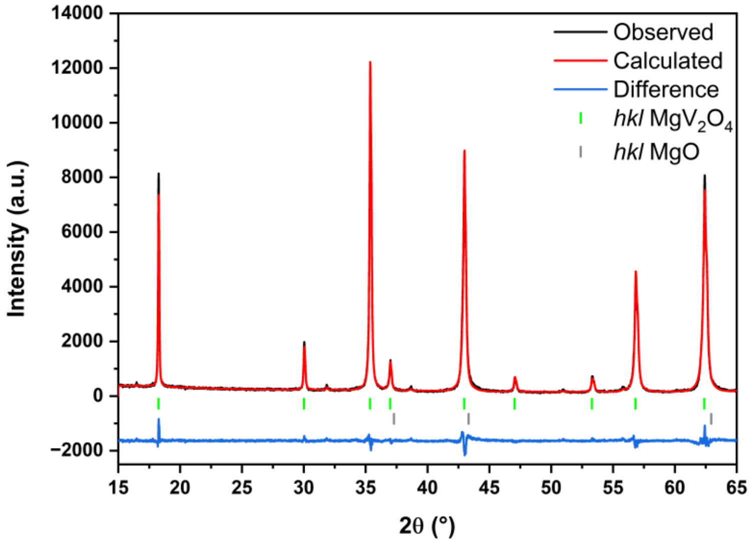

Figure S2: PXRD pattern (black) and Rietveld refinement (red) of MgV_2O_4 prepared mechanochemically at 300 rpm. Green ticks indicate the Bragg positions of MgV_2O_4 ($Fd\bar{3}m$), gray ones of MgO ($Fm\bar{3}m$).

Table S1: Refinement parameters for the 300 rpm sample using both stoichiometric MgV_2O_4 and MgO. The background polynomial is set to the 15th degree.

Phase	wt%	*a* (pm)	cry size (nm)	strain (%)	R_{wp}	GOF	composition
MgO	19	421.47(1)	32(2)	0.07(1)	7.20	1.37	$Mg_{2.10}V_2O_{5.10}$
MgV_2O_4	81	841.11(1)	>500	0.06(1)			

2.3 500 rpm

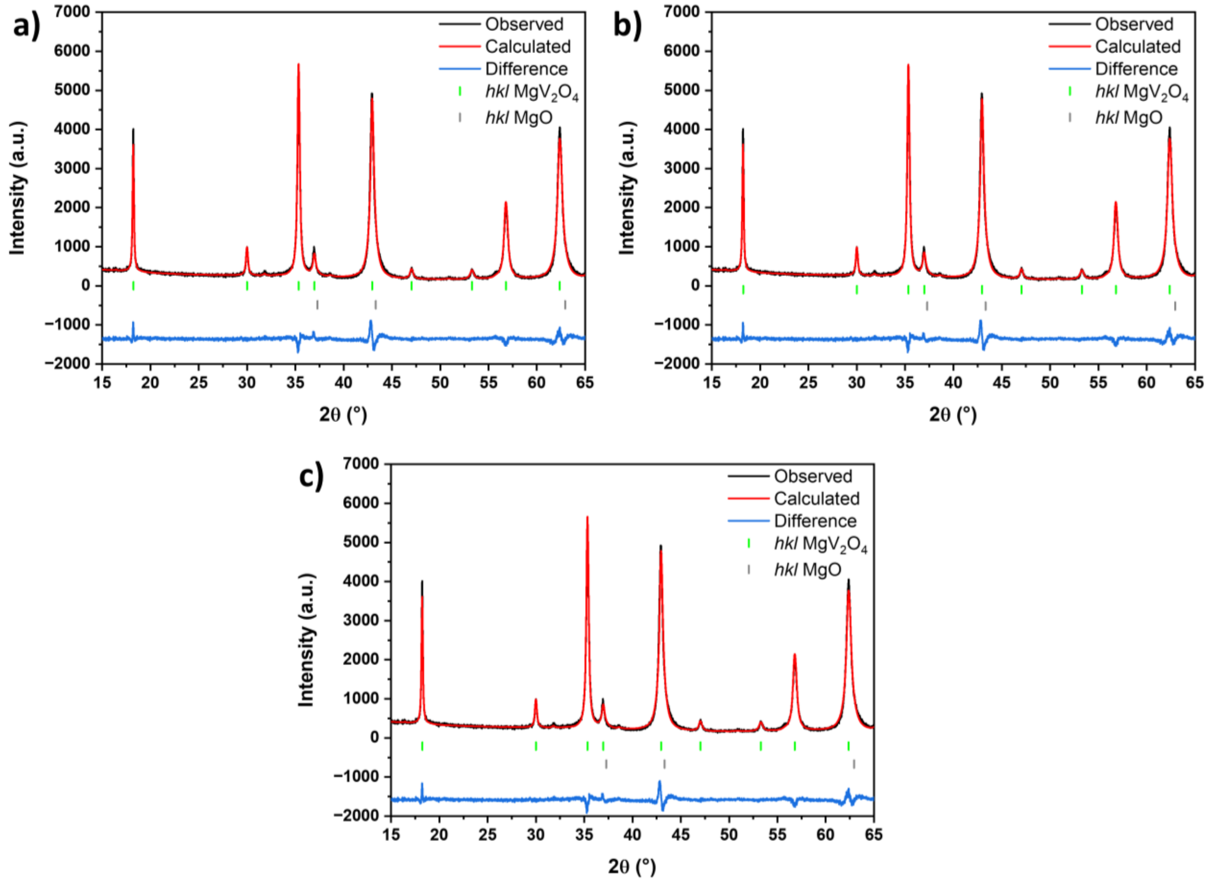

Figure S3: Different Rietveld refinements performed for MgV_2O_4 at 500 rpm, including (a) the use of stoichiometric MgV_2O_4 and MgO and using MgV_2O_4, while also allowing an insertion of (b) V(III) or (c) V(II) into MgO. In all figures, the experimental diffraction pattern is shown in black, the Rietveld refinement in red and the difference between both in blue. Green ticks indicate the Bragg positions of MgV_2O_4 ($Fd\bar{3}m$), gray ones of MgO ($Fm\bar{3}m$).

Table S2: Refinement parameters for the 500 rpm sample using both stoichiometric MgV_2O_4 and MgO, stoichiometric MgV_2O_4 and $Mg_{1-3x}V_{2x}O$ (V(III) insertion into MgO) and non-stoichiometric MgV_2O_4 and $Mg_{1-x}V_xO$ (V(II) insertion into MgO). The background polynomial is set to the 15th degree.

Phase	wt%	*a* (pm)	cry size (nm)	strain (%)	R_{wp}	GOF	composition
MgO	38	420.34(2)	16(1)	0.25(2)	7.34	1.41	$Mg_{3.93}V_2O_{6.93}$
MgV_2O_4	62	841.77(2)	222(24)	0.14(1)			
$Mg_{0.61}V_{0.26}O$	38	420.34(2)	16(1)	0.26(2)	7.21	1.40	$Mg_{1.94}V_2O_{4.94}$
MgV_2O_4	62	841.75(2)	239(28)	0.14(1)			
$Mg_{0.89}V_{0.11}O$	37	420.35(2)	16(1)	0.26(2)	7.22	1.40	$Mg_{2.97}V_2O_{5.84}$
MgV_2O_4	63	841.75(2)	237(27)	0.14(1)			

2.4 700 rpm

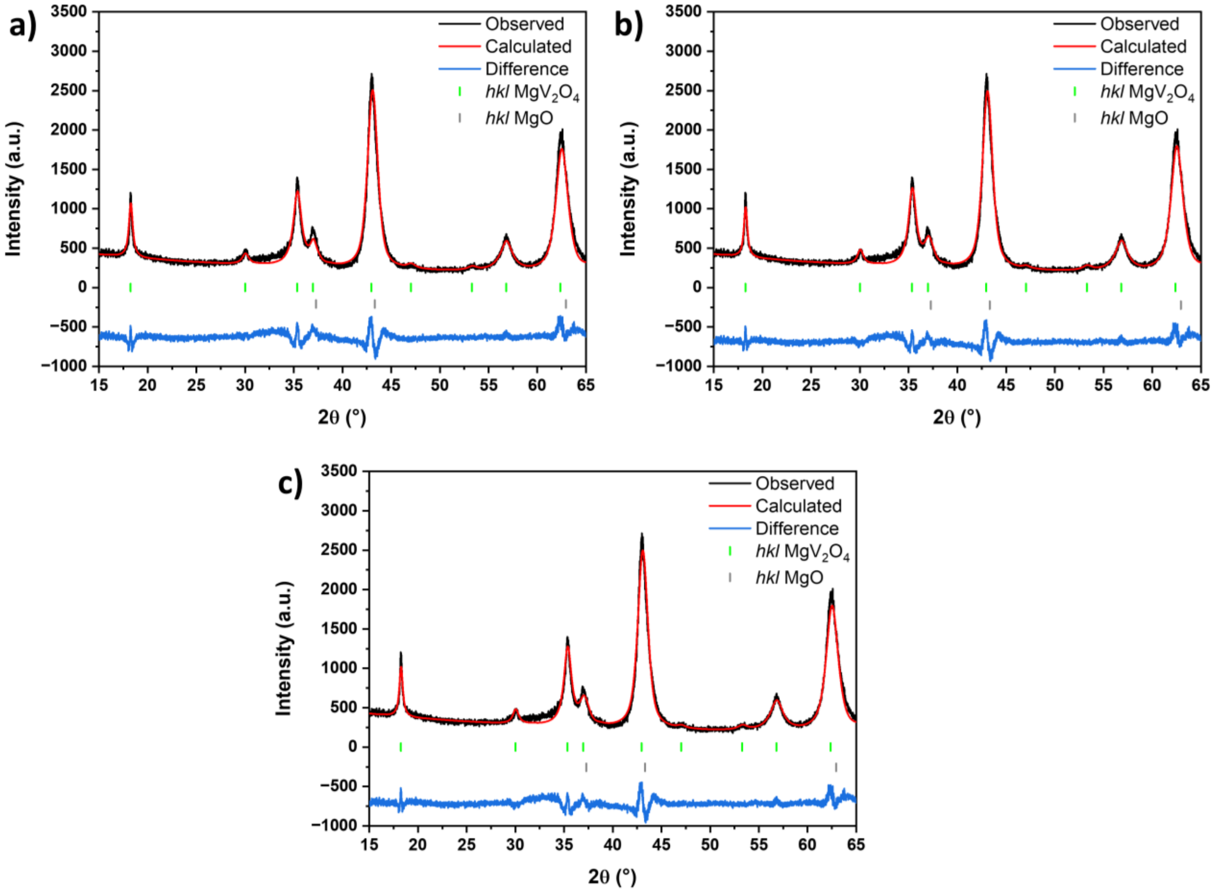

Figure S4: Different Rietveld refinements performed for MgV_2O_4 at 700 rpm, including (a) the use of stoichiometric MgV_2O_4 and MgO and using MgV_2O_4, while also allowing an insertion of (b) V(III) or (c) V(II) into MgO. In all figures, the experimental diffraction pattern is shown in black, the Rietveld refinement in red and the difference between both in blue. Green ticks indicate the Bragg positions of MgV_2O_4 ($Fd\bar{3}m$), gray ones of MgO ($Fm\bar{3}m$).

Table S3: Refinement parameters for the 700 rpm sample using both stoichiometric MgV_2O_4 and MgO, stoichiometric MgV_2O_4 and $Mg_{1-3x}V_{2x}O$ (V(III) insertion into MgO) and non-stoichiometric MgV_2O_4 and $Mg_{1-x}V_xO$ (V(II) insertion into MgO). The background polynomial is set to the 5th degree.

Phase	wt%	*a* (pm)	cry size (nm)	strain (%)	R_{wp}	GOF	composition
MgO	61	419.80(3)	13(2)	0.48(3)	8.36	1.62	$Mg_{8.25}V_2O_{11.25}$
MgV_2O_4	39	841.91(9)	16(1)	0.35(1)			
$Mg_{0.39}V_{0.40}O$	60	419.76(3)	13(2)	0.48(3)	8.14	1.58	$Mg_{1.52}V_2O_{4.56}$
MgV_2O_4	40	841.71(9)	24(2)	0.39(1)			
$Mg_{0.77}V_{0.23}O$	55	419.77(3)	14(2)	0.49(3)	8.07	1.57	$Mg_{3.81}V_2O_{6.64}$
$Mg_{1.51}V_{1.49}O_4$	45	841.69(9)	26(2)	0.40(1)			

3 Mechanochemical Stability of MgV_2O_4

To investigate the mechanochemical stability of MgV_2O_4, MgV_2O_4 prepared from V_2O_5 and Mg at 700 rpm in 20 min is further ball milled at 700 rpm. After only 30 min, reflections of MgV_2O_4 are barely visible anymore, showing its low mechanochemical stability. In addition to the decreasing MgV_2O_4 reflections, new reflections at ~31.5° and 48.3° 2θ are observable, which can be attributed to WC with space group ($P\bar{6}m2$) originating from abrasion of the WC milling material. The original sample prepared in 20 min of milling exhibits a low abrasion of WC with weight percentage of tungsten smaller than 0.3 wt%, as determined by ICP-MS measurements.

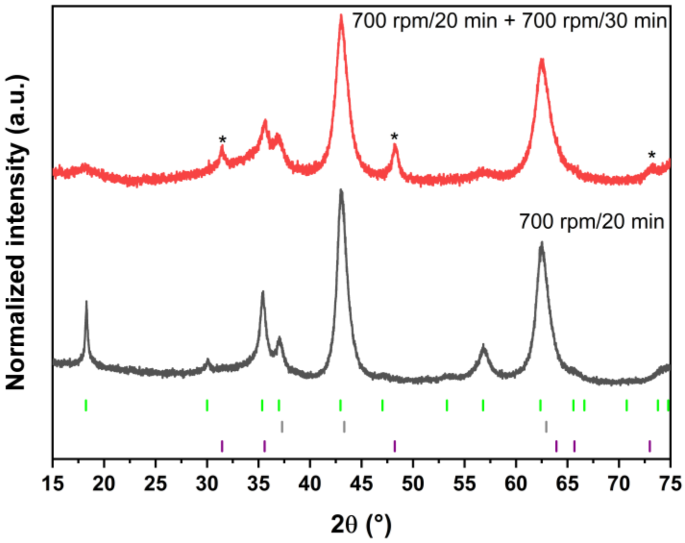

Figure S5: Normalized PXRD patterns of MgV_2O_4 prepared mechanochemically from V_2O_5 and Mg in 20 min at 500 (black) and 700 rpm (blue) and after subsequent ball milling at 700 rpm for 30 min (red, green). Green ticks indicate the Bragg positions of MgV_2O_4 ($Fd\bar{3}m$), gray ones of MgO ($Fm\bar{3}m$), and purple ones of WC ($P\bar{6}m2$). Asterisks mark the reflections of WC.

4 Simulation of Electron Paramagnetic Resonance Spectra

All spectra were recorded at a temperature of 5 K using a microwave power of 0.2 mW and magnetic field modulation amplitude set at 0.5 G with a frequency of 100 kHz.

For all optimizations, the Nelder-Mead simplex optimization algorithm was used together with the function *pepper* of EasySpin[3] with a convergence criterium of 10^{-5}.

Table S4: Simulation parameters for the 300 rpm sample using EasySpin (S = 1/2):

Simulation Parameter	Value
g-factor	$g_\perp$ = 1.940 ± 0.003, $g_{\|}$ = 1.989 ± 0.001
g-Strain	g-Strain$_\perp$ = 0.103 ± 0.06, g-Strain$_{\|}$ = 0.005 ± 0.047
Hyperfine tensor A	$A_\perp$ = 119.80 ± 3.29 MHz, $A_{\|}$ = 226.68 ± 1.46 MHz
A-Strain	A-Strain$_\perp$ = 0 MHz, A-Strain$_{\|}$ = 2.34 ± 3.46 MHz
Gaussian linewidth	0.79 ± 0.46 mT

Table S5: Simulation parameters for the 700 rpm / 50 min sample EasySpin (S = 1/2):

Simulation Parameter	Value
g-factor	$g_\perp$ = 1.977 ± 0.001, $g_{\|}$ = 1.951 ± 0.001
g-Strain	g-Strain$_\perp$ = 0, g-Strain$_{\|}$ = 0
Hyperfine tensor A	$A_\perp$ = 158.03 ± 1.34 MHz, $A_{\|}$ = 490.03 ± 2.51 MHz
A-Strain	A-Strain$_\perp$ = 20.01 ± 8.7 MHz, A-Strain$_{\|}$ = 50.02 ± 7.7 MHz
Gaussian linewidth	4.24 ± 0.20 mT

5 Rietveld refinement after MgO removal

Table S6: Phase percentage obtained by Rietveld refinement depending on the washing procedure.

Sample	MgV_2O_4 (wt%)	MgO wt(%)
300 rpm - unwashed	81	19
300 rpm - washed	98	2
500 rpm - unwashed	62	38
500 rpm - washed	95	5
700 rpm - unwashed	45	55
700 rpm - washed	79	21

Table S7: Refinement parameters for the washed 700 rpm sample using both stoichiometric MgV_2O_4 and MgO, stoichiometric MgV_2O_4 and $Mg_{1-3x}V_{2x}O$ (V(III) insertion into MgO) and non-stoichiometric MgV_2O_4 and $Mg_{1-x}V_xO$ (V(II) insertion into MgO). The background is set to 5.

Phase	wt%	a (pm)	cry size (nm)	strain (%)	R_{wp}	GOF	composition
MgO	25	418.75(3)	13(1)	0.24(1)	7.95	1.52	$Mg_{2.6}V_2O_{5.7}$
MgV_2O_4	75	841.22(9)	58(3)	0.27(1)			
$Mg_{0.29}V_{0.47}O$	25	418.78(3)	11(1)	0.23(1)	7.89	1.51	$Mg_{1.06}V_2O_{4.06}$
MgV_2O_4	75	841.20(3)	61(4)	0.27(1)			
$Mg_{0.77}V_{0.23}O$	24	418.75(3)	12(1)	0.23(1)	7.83	1.50	$Mg_{1.78}V_2O_{4.70}$
$Mg_{1.18}V_{1.82}O_4$	76	841.19(6)	63(4)	0.27(1)			

6 EPR spectra after washing

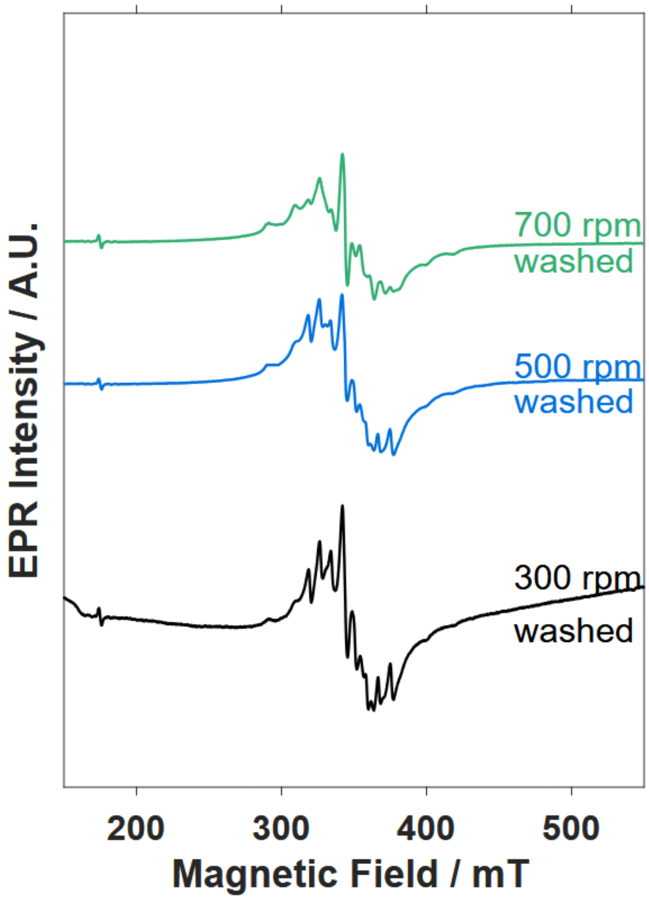

Figure S6: (a) Overview of continuous wave (CW) EPR spectra at 5 K of MgV_2O_4 prepared in 20 min at 300 (black), 500 (blue) and 700 rpm (red), followed by acid washing.

7 Determination of the Mg to V ratio by ICP-MS analysis

Table S8: Ratio of magnesium to vanadium determined by ICP-MS measurements of unwashed and washed samples prepared by milling V_2O_5 with 2 eq. Mg for 20 min for different rotational speeds.

Sample	Ratio Mg : V
300 rpm, unwashed	0.93 : 1
300 rpm, washed	0.66 : 1
500 rpm, unwashed	0.98 : 1
500 rpm, washed	0.59 : 1
700 rpm, unwashed	0.96 : 1
700 rpm, washed	0.68 : 1

8 Results of SEM measurements

While SEM images of the sample prepared at 300 pm reveal the presence of mainly micrometer-sized particles along with a few smaller ones, harsher milling conditions lead to a decrease of the particle size towards the nanometer range. In addition, the formation of agglomerates is observed.

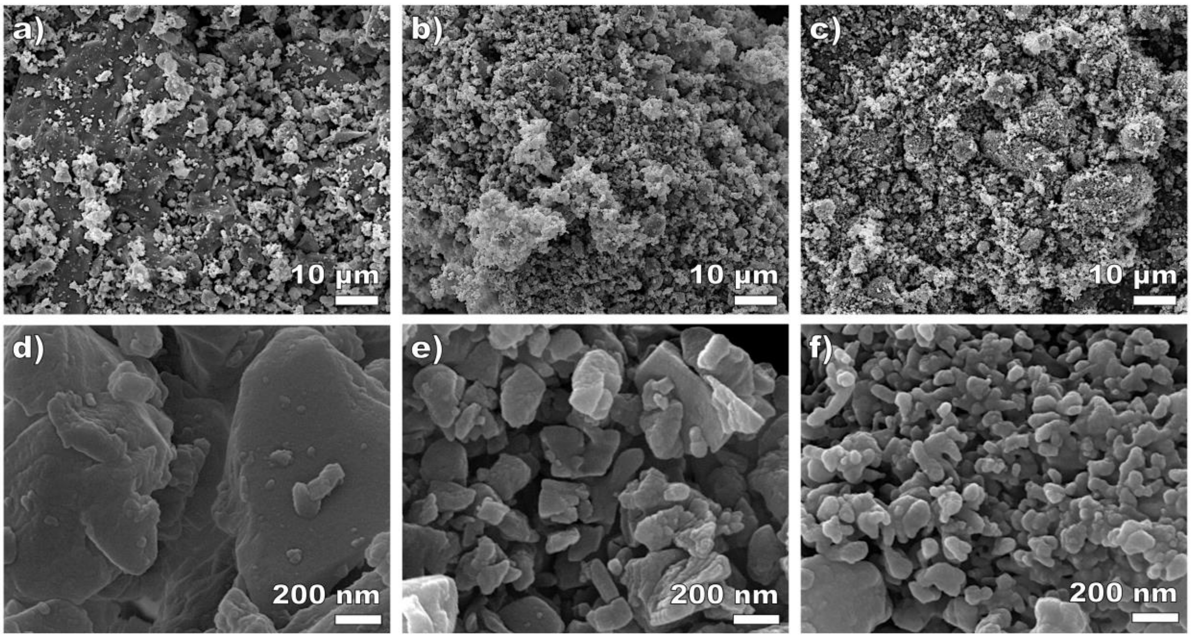

Figure S7: SEM photographs of samples prepared at (a) and (d) 300 rpm, (b) and (e) 500 rpm, as well as (c) and (f) 700 rpm. For better visualization, note the different magnification factors of the top and bottom rows for the same samples.

9 Electrochemical Impedance Spectroscopy

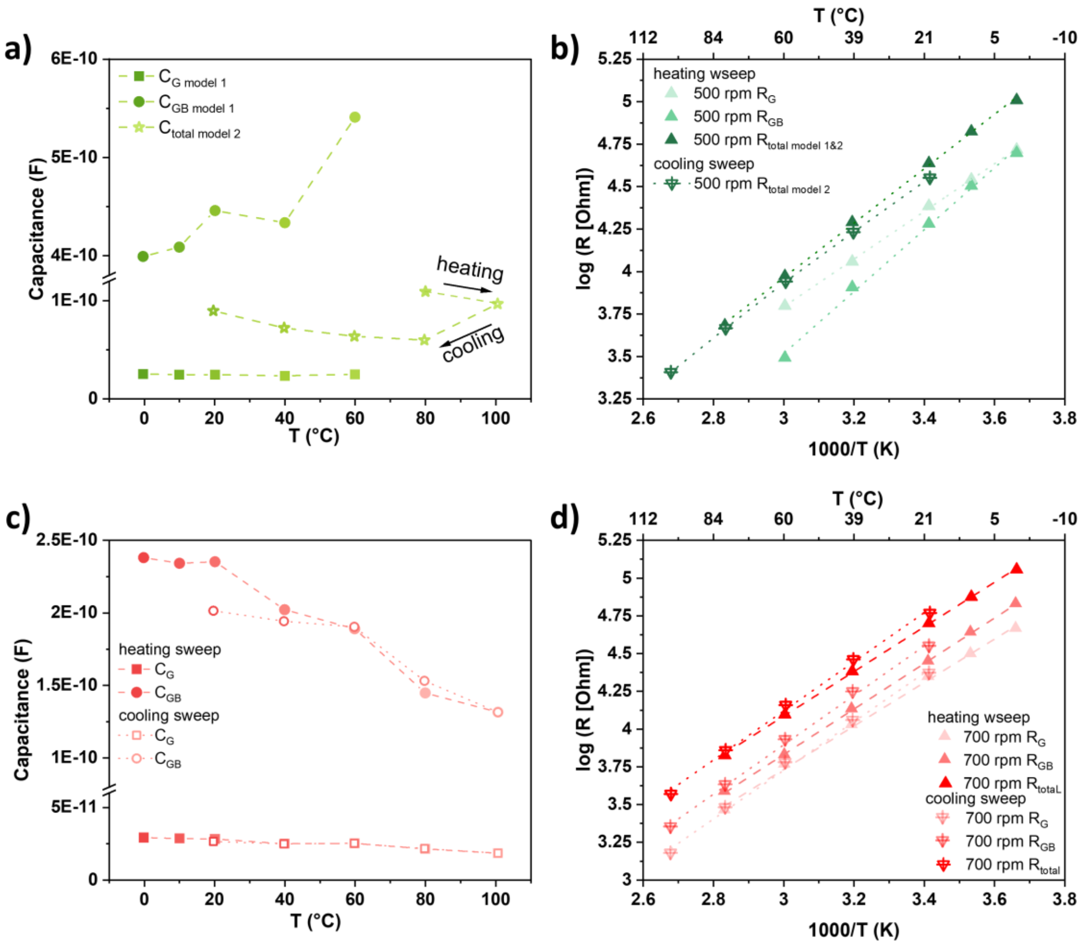

Figure S8: (a) Temperature-dependent bulk(grain) capacitance and grain boundary capacitance for 500 rpm-milled sample; (b) temperature-dependent bulk(grain), grain boundary and total resistance for 500 rpm-milled sample; (c) temperature-dependent bulk(grain) capacitance and grain boundary capacitance for 700 rpm-milled sample and (d) corresponding bulk(grain), grain boundary and total resistance.

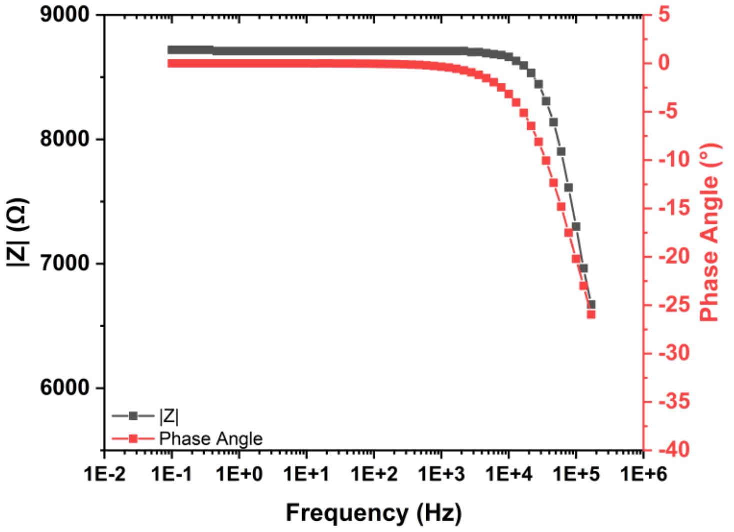

Figure S9: Bode plot of 500 rpm-milled sample at 60 °C on cooling sweep.

10 References

(1) Bruker AXS Inc.: *DIFFRAC.SUITE, Topas 5 User Manual*, Karlsruhe (Germany), **2014**.

(2) Scarlett, N. V. Y.; Madsen, I. C. Quantification of phases with partial or no known crystal structures. *Powder Diffr.* **2006**, *21*, 278-284.

(3) Stoll, S.; Schweiger, A. EasySpin, a comprehensive software package for spectral simulation and analysis in EPR. *J. Magn. Reson.* **2006**, *178*, 42-55.
